# Concentration-dependent Dual Effects of Hydrogen Peroxide on Insulin Signal Transduction in H4IIEC Hepatocytes

**DOI:** 10.1371/journal.pone.0027401

**Published:** 2011-11-15

**Authors:** Satoshi Iwakami, Hirofumi Misu, Takashi Takeda, Makoto Sugimori, Seiichi Matsugo, Shuichi Kaneko, Toshinari Takamura

**Affiliations:** 1 Department of Disease Control and Homeostasis, Kanazawa University Graduate School of Medical Science, Kanazawa, Japan; 2 Division of Material Engineering, Graduate School of Natural Science and Technology, Kanazawa University, Kanazawa, Japan; 3 College of Science and Engineering, School of Natural System Bioengineering Course, Kanazawa University, Kanazawa, Japan; Roswell Park Cancer Institute, United States of America

## Abstract

**Background:**

Oxidative stress induced by the accumulation of reactive oxygen species (ROS) has a causal role in the development of insulin resistance, whereas ROS themselves function as intracellular second messengers that promote insulin signal transduction. ROS can act both positively and negatively on insulin signaling, but the molecular mechanisms controlling these dual actions of ROS are not fully understood.

**Methodology/Principal Findings:**

Here, we directly treated H4IIEC hepatocytes with hydrogen peroxide (H2O2), a representative membrane-permeable oxidant and the most abundant ROS in cells, to identify the key factors determining whether ROS impair or enhance intracellular insulin signaling. Treatment with high concentrations of H2O2 (25–50 µM) for 3 h reduced insulin-stimulated Akt phosphorylation, and increased the phosphorylation of both JNK and its substrate c-Jun. In contrast, lower concentrations of H2O2 (5–10 µM) enhanced insulin-stimulated phosphorylation of Akt. Moreover, lower concentrations suppressed PTP1B activity, suggesting that JNK and phosphatases such as PTP1B may play roles in determining the thresholds for the diametrical effects of H2O2 on cellular insulin signaling. Pretreatment with antioxidant N-acetyl-L-cysteine (10 mM) canceled the signal-promoting action of low H2O2 (5 µM), and it canceled out further impairment of insulin of insulin signaling induced by high H_2_O_2_ (25 µM).

**Conclusions/Significance:**

Our results demonstrate that depending on its concentration, H2O2 can have the positive or negative effect on insulin signal transduction in H4IIEC hepatocytes, suggesting that the concentration of intracellular ROS may be a major factor in determining whether ROS impair or enhance insulin signaling.

## Introduction

Insulin resistance is an underlying problem in people with type 2 diabetes and metabolic syndrome [1]. In an insulin-resistant state, impaired insulin action promotes hepatic glucose production and reduces the uptake of glucose by peripheral tissues, resulting in systemic hyperglycemia. In addition to type 2 diabetes and metabolic syndrome, the development of various other diseases such as non-alcoholic steatohepatitis [2] and atherosclerosis [3] involves insulin resistance. It is commonly assumed that combating insulin resistance is a viable therapeutic strategy in several kinds of diseases, although the molecular mechanisms underlying insulin resistance are not fully understood.

Oxidative stress induced by the accumulation of reactive oxygen species (ROS) has a causal role in the development of insulin resistance. Using models in which cells were treated with tumor-necrosis factor α and glucocorticoids, Houstis et al. showed that increased ROS levels are an important trigger for insulin resistance in numerous contexts [4]. Activation of stress kinases such as C-Jun N-terminal kinase (JNK) and IκB kinase contributes to insulin resistance associated with oxidative stress [5]. In a previous report, we demonstrated that treatment with palmitate, a C16∶0 saturated fatty acid, induces insulin resistance in H4IIEC hepatocytes by stimulating the generation of ROS in the mitochondria and thereby, the activation of JNK [6]. The administration of antioxidants such as N-acetylcysteine and α-tocopherol partially rescued cells from palmitate-induced insulin resistance, suggesting that antioxidative therapy may be useful in attenuating insulin resistance in patients with type 2 diabetes or metabolic syndrome.

A growing body of evidence suggests that ROS function as intracellular second messengers to promote signaling by hormones, including insulin. Goldstein et al. have shown that insulin-induced endogenous hydrogen peroxide enhances proximal and distal insulin signaling, at least partly through the oxidative inhibition of protein tyrosine phosphatase 1B (PTP1B), which negatively regulates insulin action [7]. More recently, Loh et al. reported that mice lacking glutathione peroxidase 1 (Gpx1), a key enzyme involved in the removal of ROS, are protected from high-fat diet-induced insulin resistance, providing causal evidence for the enhancement of insulin signaling by ROS *in vivo* [8]. These early reports indicate that ROS can act both positively and negatively on insulin signaling. However, the molecular mechanisms regulating the dual actions of ROS on insulin signaling are not fully understood.

In the current study, to identify the key factors determining whether ROS impair or enhance intracellular insulin signaling, we directly treated H4IIEC hepatocytes with hydrogen peroxide (H_2_O_2_), a representative membrane-permeable oxidant and the most abundant ROS in the cell. Our results demonstrate that H_2_O_2_ has dual effects on insulin signal transduction in H4IIEC hepatocytes and that these roles depend on the H_2_O_2_ concentration used, suggesting that the intracellular concentration of ROS themselves may be a major factor in determining whether ROS impair or enhance insulin signaling.

## Results

### Time course of extracellular H_2_O_2_ concentration following its administration to H4IIEC hepatocytes

**Figure 1:**
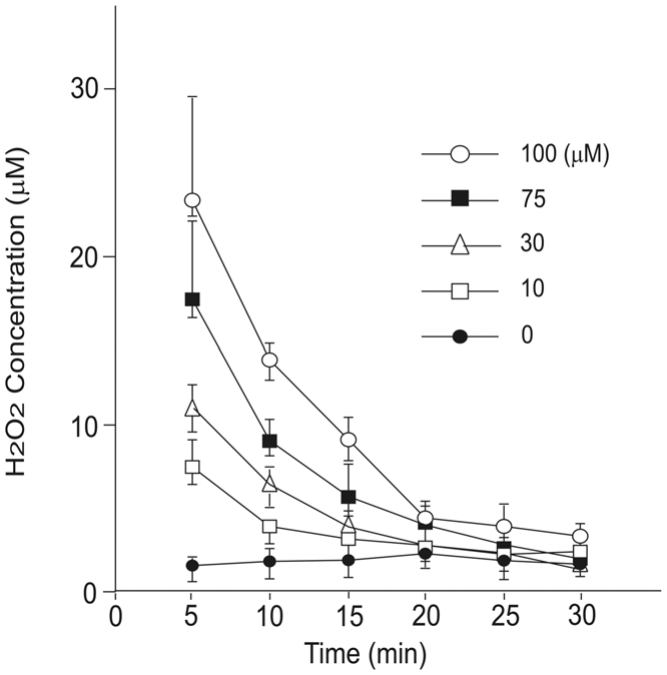
Time course of extracellular H2O2 concentration following its administration to H4IIEC hepatocytes. H4IIEC cells were incubated with the indicated concentrations of H2O2 for the indicated periods of time, and then the concentration of H2O2 in the culture medium was measured by ferrous oxidation of xylenol orange (FOX) assay.

We treated H4IIEC hepatocytes with H_2_O_2_, a representative membrane-permeable ROS. Exogenous H_2_O_2_ is time-dependently reduced and neutralized by intracellular antioxidant enzymes. To assess how long the H_2_O_2_ remained effective, we measured H_2_O_2_ concentrations in the culture medium over a specific time course after its administration (Fig. 1). The H_2_O_2_ concentration in the culture medium returned to basal level within 30 min of its administration to H4IIEC hepatocytes. Therefore, when cells were treated with H_2_O_2_ for a total of 3 h in subsequent experiments, the H_2_O_2_-containing medium was replaced every 30 min.

### Dual effects of H_2_O_2_ on insulin-stimulated phosphorylation of Akt and GSK-3

**Figure 2:**
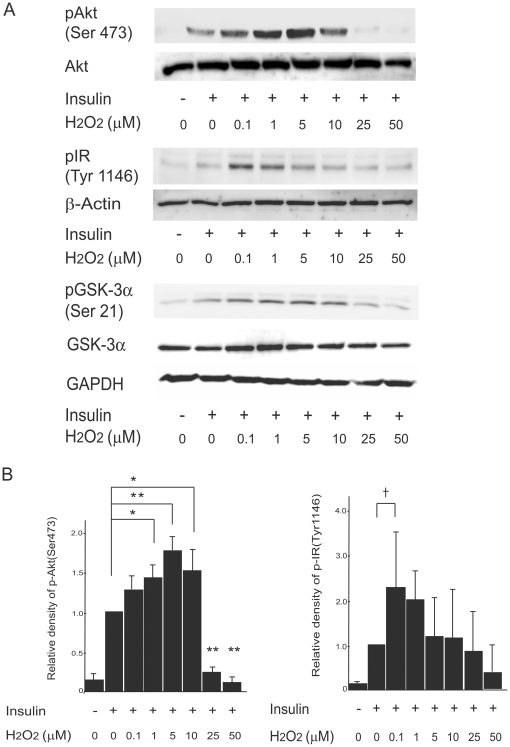
Effects of H2O2 on insulin-stimulated tyrosine phosphorylation of the IR and serine phosphorylation of Akt and GSK-3 in H4IIEC hepatocytes. (A) H4IIEC cells were serum-starved overnight and then treated with the indicated concentrations of H2O2 for total 3 h. Then, the cells were stimulated with insulin (1 ng/mL) for 15 min. Total cell lysates were resolved by SDS-PAGE, transferred to a PVDF membrane, and probed using the indicated antibodies. Signals were detected by chemiluminescence. Representative blots are shown. (B) Quantitative data from densitometric analysis of blots from three (p-IR) and four (p-Akt) independent experiments, respectively. Relative density is mean fold increase over control ± S.E.M. * *p*<0.05, versus treatment with insulin alone; ** *p*<0.01, versus treatment with insulin alone.

We examined the effects of H_2_O_2_ on insulin signaling in H4IIEC hepatocytes (Fig. 2). H4IIEC3 cells were treated with a range of H_2_O_2_ concentrations for 3 h and then stimulated with insulin for 15 min. As expected, insulin-stimulated serine phosphorylation of Akt and GSK-3α was inhibited at high concentrations of H_2_O_2_. Insulin-stimulated tyrosine phosphorylation of the insulin receptor (IR) was unaffected by high H_2_O_2_ concentrations. In contrast, lower concentrations of H_2_O_2_ enhanced insulin-stimulated phosphorylation of Akt and GSK-3α, without affecting the overall levels of the proteins. We found that 5–10 µM were the concentrations of which H_2_O_2_ certainly promotes insulin-induced Akt phosphorylation in our cell lines. Thus, we used 5–10 µM of H_2_O_2_ to certainly increase insulin signaling in the following experiments.

### Effects of H_2_O_2_ treatment on the expression of genes encoding antioxidant enzymes

**Figure 3:**
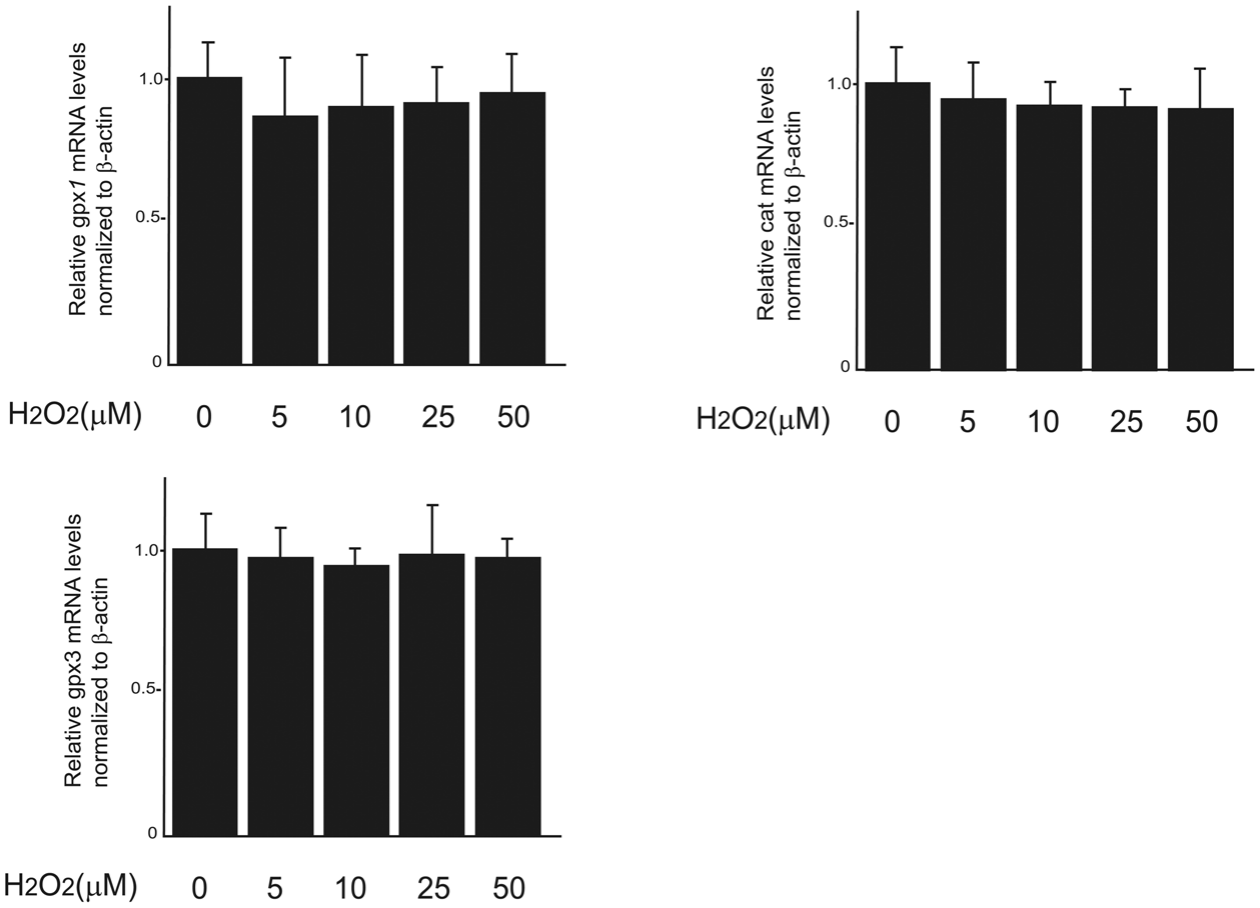
Effects of H_2_O_2_ on the expression of mRNAs encoding antioxidative enzymes in H4IIEC hepatocytes. H4IIEC cells were serum-starved overnight and treated with the indicated concentrations of H_2_O_2_ for 3 h. Levels of the mRNAs encoding GPX1, GPX3, and catalase were measured by real-time-PCR. Values represent the mean ± S.D (*n* = 6).

We hypothesized that weak oxidative stress caused by low concentrations of H_2_O_2_ may trigger a preconditioning response by inducing antioxidant enzymes such as catalase and Gpx. We assayed the expression of the genes encoding catalase, Gpx1, and Gpx3 in H4IIEC hepatocytes 3 h after treatment with various concentrations of H_2_O_2_ (Fig. 3). Treatment with H_2_O_2_ at concentrations in the range 5–50 µM did not alter the expression of any of these three genes.

### Attenuation of PTP1B activity contributes to H_2_O_2_-induced enhancement of insulin signaling

**Figure 4:**
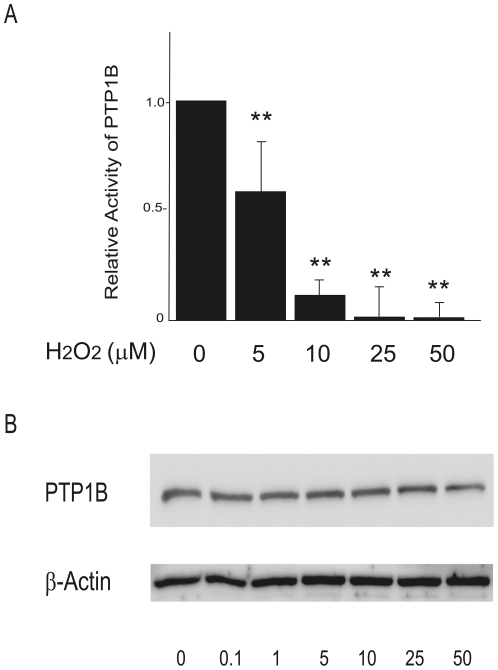
Effects of H2O2 on PTP1B activity in H4IIEC hepatocytes. (A) H4IIEC cells were serum-starved overnight and then treated with the indicated concentrations of H2O2. After snap-freezing in liquid N2, cells were disrupted through scraping into ice-cold RIPA buffer containing a protease inhibitor cocktail, followed by brief sonication and clearing of the resulting lysates by centrifugation at 15,000 rpm for 15 min. PTP1B activity was determined using a CycLex® Protein Tyrosine Phosphatase 1B (PTP1B) Fluorometric Assay Kit. Fluorescence values from three independent experiments were normalized to total protein concentrations and are expressed as mean fold increases over control ± S.D. ** *p*<0.01, versus untreated control. (B) Protein levels of PTP1B were measured by Western blotting.

**Figure 5:**
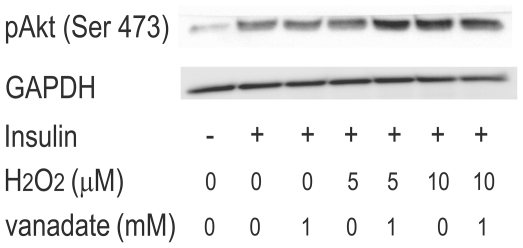
Effects of vanadate on insulin-stimulated Akt phosphorylation in H4IIEC hepatocytes. H4IIEC cells were serum-starved overnight. Then the cells were treated with the indicated concentrations of H_2_O_2_ for 3 h. Then, the cells were treated with 1mM of sodium orthovanadate for 30 min, and stimulated with insulin (1 ng/mL) for 15 min. Total cell lysates were resolved by SDS-PAGE, transferred to a PVDF membrane, and probed using the indicated antibodies.

To clarify the mechanism by which low concentrations of H_2_O_2_ enhance insulin signaling, we measured the activity of PTP1B, a negative regulator of insulin signaling, in H_2_O_2_-treated cells. PTP1B activity was dose-dependently suppressed by H_2_O_2_ (Fig. 4A), even at H_2_O_2_ concentrations as low as 5 µM. Overall PTP1B protein levels were unaffected by H_2_O_2_ treatment (Fig 4B). To further determine whether phosphatases mediate the insulin-promotive effect induced by low concentrations of H_2_O_2_, we treated the cells with vanadate, a non-specific inhibitor for phosphatases such as PTP1B (Fig. 5). Coadministration with vanadate increased insulin-stimulated Akt phosphorylation in the cells treated with 5 µM of H_2_O_2_. However, it did not further increase insulin-stimulated Akt phosphorylation in the presence of 10 µM of H_2_O_2_ (Fig. 5).

### Activation of JNK contributes to H_2_O_2_-induced insulin resistance

**Figure 6:**
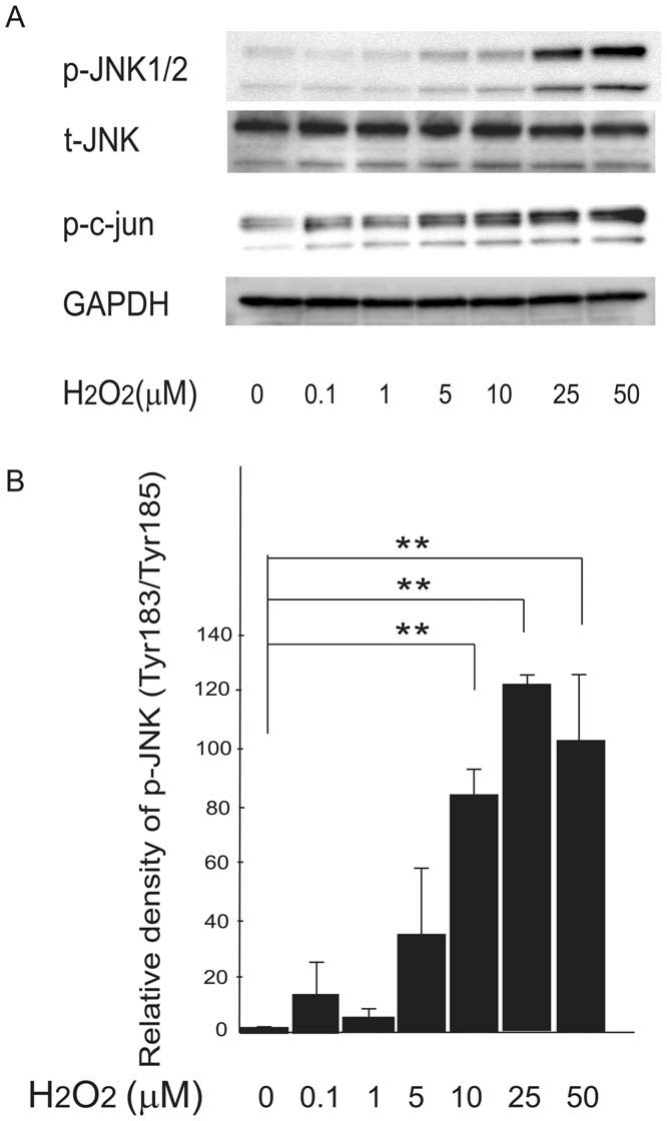
Effects of H2O2 on JNK activation in H4IIEC hepatocytes. (A) H4IIEC cells were serum-starved overnight and then treated with the indicated concentrations of H2O2. Total cell lysates were resolved by SDS-PAGE, transferred to a PVDF membrane, and probed with the indicated antibodies. Signals were detected chemiluminescence. Representative blots are shown. (B) Quantitative data from densitometric analysis of p-JNK signals from three independent experiments were normalized to the values for total JNK and are expressed as mean fold increases over control ± S.D. ** *p*<0.01, versus untreated control.

**Figure 7:**
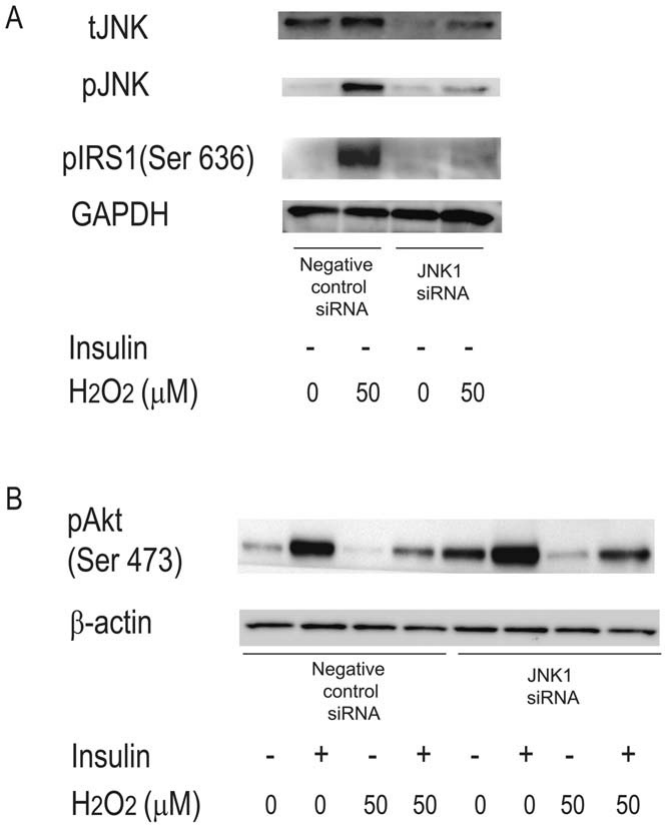
Effects of JNK knockdown on H_2_O_2_-induced alterations of insulin signal transduction in H4IIEC hepatocytes. H4IIEC cells were transfected with siRNA specific to JNK1 or negative control. 24 h later, the cells were serum-starved overnight. Then the cells were treated with the indicated concentrations of H_2_O_2_ for 3 h, and stimulated with insulin (1 ng/mL) for 15 min. Total cell lysates were resolved by SDS-PAGE, transferred to a PVDF membrane, and probed using the indicated antibodies. (A) Protein levels of total JNK, phosphorylated INK, and phosphorylated serine residue of IRS-1 in the cells transfected with JNK. (B) Protein levels of phosphorylated Akt in the cells stimulated with insulin.

To examine the mechanism by which high concentrations of H_2_O_2_ impair insulin signaling, we measured the levels of phospho-JNK in H_2_O_2_-treated cells. JNK is a stress-activated protein kinase that phosphorylates IRS-1 and -2 at serine residues in response to increases in cellular ROS levels. JNK-induced serine phosphorylation of IRS impairs IRS tyrosine phosphorylation, resulting in the inhibition of insulin receptor-mediated signaling. Treatment with H_2_O_2_ increased the phosphorylation of both JNK and its direct target, c-Jun dose-dependently (Fig. 6). To further determine whether JNK mediates insulin resistance induced by high concentrations of H_2_O_2_, we transfected H4IIEC hepatocytes with siRNA specific for JNK1 (Fig. 7). Knockdown of JNK partly suppressed serine phosphorylation of IRS-1 induced by high concentrations of H_2_O_2_ (Figure 7A). As a result, insulin resistance induced by high concentrations of H_2_O_2_ was partly rescued by silencing of JNK (Fogiure 7B). These results suggest that high concentrations of H_2_O_2_ reduce insulin-stimulated Akt phosphorylation, at least partly, by increasing the phosphorylation of JNK and serine residue of IRS-1.

### Effects of antioxidant N-acetyl-L-cysteine on H_2_O_2_-induced alterations of insulin signal transduction in H4IIEC hepatocytes

**Figure 8:**
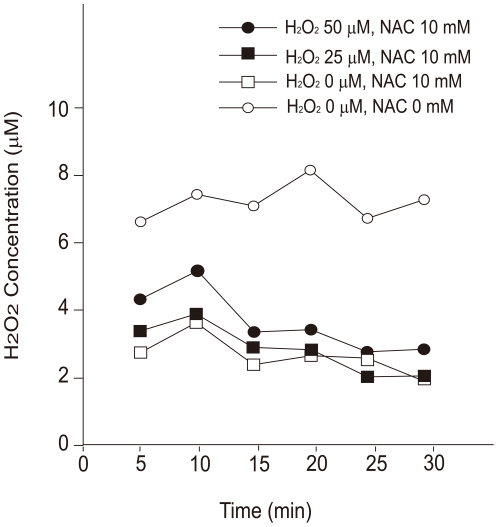
Time course of extracellular H2O2 concentration following its administration to H4IIEC hepatocytes pretreated with *N*-acetyl-L-cysteine. H4IIEC cells were serum-starved and treated with 10 mM of N-acetyl-L-cysteine overnight. Then, the cells were incubated with the indicated concentrations of H2O2 for the indicated periods of time, and the concentration of H2O2 in the culture medium was measured by ferrous oxidation of xylenol orange (FOX) assay.

**Figure 9:**
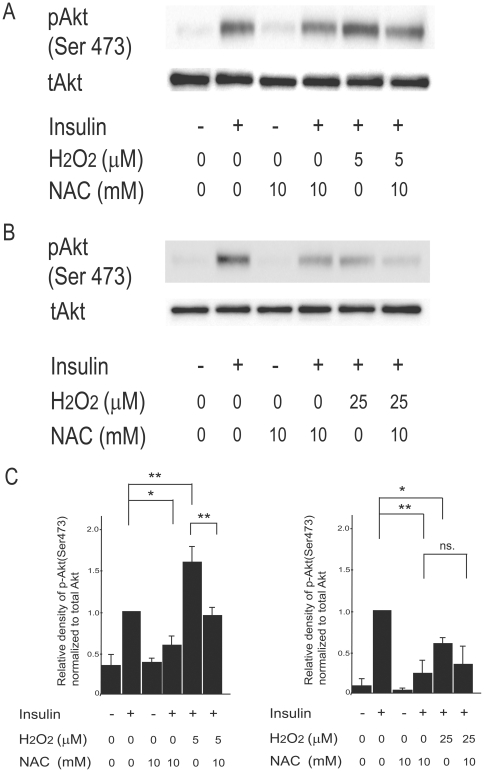
Effects of antioxidant *N*-acetyl-L-cysteine on H_2_O_2_-induced alterations of insulin signal transduction in H4IIEC hepatocytes. (A–B) H4IIEC cells were serum-starved and treated with 10 mM of N-acetyl-L-cysteine overnight. Then the cells were treated with the indicated concentrations of H_2_O_2_ for 3 h, and stimulated with insulin (1 ng/mL) for 15 min. Total cell lysates were resolved by SDS-PAGE, transferred to a PVDF membrane, and probed using the indicated antibodies. (C) Quantitative data from densitometric analysis of p-Akt signals from three independent experiments were normalized to the values for total Akt and are expressed as mean fold increases over control ± S.E.M. . * *p*<0.05, versus treatment with insulin alone; ** *p*<0.01, versus treatment with insulin alone.

Next, we determined whether antioxidant N-acetyl-L-cysteine (NAC) attenuates the dual actions of H_2_O_2_ on insulin signal transduction. We pretreated H4IIEC hepatocytes with 10 mM of NAC, which corresponds to the maximum concentration of NAC commonly used in *in vitro* experiments [6]. Pretreatment with NAC decreased H_2_O_2_ concentrations in the culture medium of the cells treated with H_2_O_2_ (Fig. 8). NAC at the concentration of 10 mM was enough to quench H_2_O_2_ at up to 50 µM. NAC canceled the signal-promoting action of low concentrations of H_2_O_2_ (Fig. 9A). In addition, although NAC impaired insulin-stimulated phosphorylation of Akt in the absence of H_2_O_2_, it canceled out further impairment of insulin signaling induced by 25 µM of H_2_O_2_ (Fig. 9B).

## Discussion

In the current study, we used the membrane-permeable oxidant H_2_O_2_ to demonstrate that ROS can exert either positive or negative effects on insulin signal transduction, depending on the ROS concentration. Our data suggest that the overall concentration of ROS, irrespective of the subcellular compartments in which they act, determines whether they enhance or impair insulin signal transduction.

The observed negative effects of high H_2_O_2_ concentrations on insulin signal transduction are consistent with our previous report, which showed that mitochondria-derived ROS induce insulin resistance in H4IIEC hepatocytes treated with palmitate by activating JNK [6]. Many lines of evidence suggest that chronic accumulation of ROS has a causal role in the development of insulin resistance [4], [5]. The activation of stress kinases such as JNK is thought to play a central role in the development of ROS-associated insulin resistance. The upstream molecules that regulate JNK phosphorylation in response to increases in cellular ROS levels include thioredoxin (TRX) and apoptosis signal-regulating kinase 1 (ASK1) [9], [10]. ROS oxidizes TRX and consequently removes it from pre-existing TRX-ASK1 complexes, leading to the activation of ASK1 and JNK signaling. The results of our previous *in vitro* study indicated that using antioxidants to remove ROS and consequently suppress JNK activation has the potential to improve insulin sensitivity [6]. To date, however, the larger clinical intervention trials conducted to evaluate the potential of antioxidant supplements in preventing the development of diabetes have been unable to observe any positive effects [11], [12], [13]. These conflicting findings led us to hypothesize that the complete removal of ROS from cells does not necessarily improve insulin resistance, and to pay particular attention to the dose-dependent dual actions of ROS on insulin signaling.

The most surprising finding from our study is that H_2_O_2_ can exert both positive and negative effects on insulin signaling within a relatively narrow concentration range of 5–50 µM. Banerjee et al. reported blood levels of H_2_O_2_ to be approximately 1.7±2.5 µM in healthy humans [14], suggesting that our finding of enhanced insulin signaling with 5–10 µM H_2_O_2_ reflects a physiological, rather than pharmacological, action of H_2_O_2_. H_2_O_2_ is reported to have insulin-mimetic actions in insulin target cells [15], [16]. However, in these earlier studies, cells were treated with millimolar concentrations of H_2_O_2_ for short periods of time. To our knowledge, the present study is the first to demonstrate that mild oxidative stress, such as that caused by 5–10 µM H_2_O_2_, enhances intracellular insulin-mediated signaling. Recent reports have proposed that cells repeatedly exposed to sublethal stresses can develop stress resistance and display increased survival rates, as a result of a process called hormesis. The initial evidence for this novel concept was obtained using model organisms such as nematodes [17] and rats [18]. More recently, Ristow et al. extended the concept of hormesis to humans. They showed that exercise-induced low-grade oxidative stress improves insulin sensitivity in skeletal muscle [19]. However, few reports detailing the dual effects of H_2_O_2_ on insulin signaling in insulin target cells have been published. The cellular insulin signaling might be maintained by continuous exposure of the cells to low levels of ROS, while insulin resistance develops when cellular H_2_O_2_ levels rise above a certain threshold.

We show that pretreatment with NAC had a tendency to reduce insulin signaling in both H_2_O_2_-treated and untreated cells (Fig. 9)_._ In addition, we found that the cells treated with NAC and H_2_O_2_ showed lower levels of ROS than those in the absence of H_2_O_2_ (Fig. 8), suggesting that the current concentrations of NAC had a potent anti-oxidative capacity. Goldstein et al. have reported that endogenous H_2_O_2_ production stimulated by insulin is needed for the subsequent insulin signaling by inactivating phosphatases such as PTP1B [7]. Hence, we speculate that pretreatment with NAC inhibited insulin signaling generally by removing insulin-induced endogenous H_2_O_2_ production required for the suppression of phosphatase activity due to its strong anti-oxidative capacity.

In our experimental system, it was hard to determine the minimum threshold concentrations of which H_2_O_2_ exerts promotive effects on insulin signaling, because anti-oxidative capacity in the cells seemed to vary at individual experiments depending on only a modest variation of the viability or numbers of the cells cultured. However, we found that 5–10 µM were the concentrations of which H_2_O_2_ certainly promotes insulin signal transduction in our cell lines, because these concentrations of H_2_O_2_ not only increased insulin-stimulated Akt phosphorylation reproducibly (Fig. 2), but also suppressed PTP1B activity significantly (Fig. 4). Thus, we used 5–10 µM of H_2_O_2_ to certainly increase insulin signaling in the following experiments.

Our findings indicate that the enhancement of insulin signaling by low concentrations of H_2_O_2_ is accompanied by the suppression of PTP1B activity, and is rescued by the co-administration with vanadate, a non-specific inhibitor for phosphatases. We showed that vanadate further increased insulin signaling in the cells treated with 5 µM of H_2_O_2_, but not in those treated with 10 µM of H_2_O_2_ (Fig. 5). These findings seem to be reasonable, because our results showed that 5 µM of H_2_O_2_ decreased PTP1B activity by 40%, whereas 10 µM of H_2_O_2_ decreased it by approximately 90% (Fig. 4). Thus, we speculate that co-administration with vanadate was unable to further enhance insulin signaling in the cells where PTP1B activity was almost completely suppressed by 10 µM of H_2_O_2_. These results suggest that low concentrations of H_2_O_2_ enhance insulin signaling, at least partly, by inhibiting phosphatases such as PTP1B. PTP1B, a protein tyrosine phosphatase (PTP), negatively regulates insulin signaling by dephosphorylating a tyrosine residue of the insulin receptor [20]. The structure of the active site and the low pKa of the thiol group of the invariant catalytic cysteine residue render PTP1B highly susceptible to reversible oxidation by H_2_O_2_ [21]. Oxidation of its active site cysteine residue abolishes PTP1B's nucleophilic properties, causing it to be inactivated. Goldstein et al. showed that an insulin-stimulated endogenous H_2_O_2_ burst positively regulated insulin signaling, at least partly through oxidative inhibition of PTP1B [7]. However, early papers showed that compounds of vanadium inhibit several forms of phosphatases including PTP1B [22], [23]. Thus, additional studies are needed to determine whether low concentrations of H_2_O_2_ enhance insulin signaling in a PTP1B-specific manner.

Peak IR phosphorylation was observed in the cells treated with the lower concentrations of H_2_O_2_ compared with Akt phosphorylation (Fig. 2). These results suggest that the action mechanisms of low concentrations of H_2_O_2_ are different on the phosphorylation of Akt and IR. Several kinds of phosphatases are known to inactivate insulin signaling cascade by dephosphorylating different kinds of downstream effectors of insulin. Phosphatase and tensin homolog deleted on chromosome 10 (PTEN) dephosphorylates phosphatidylinositol 3, 4, 5-trisphosphate (PIP3) to terminate PI3 kinase signaling, resulting in a selective inhibition of PI3 kinase/Akt signaling, although PTP1B dephosphorylates tyrosine residue of insulin receptor, resulting in a total inhibition of insulin signaling. PTEN is also inactivated by H_2_O_2_–induced oxidation as well as PTP1B [24]. The different sensitivities of H_2_O_2_ to the phosphorylation of Akt and IR might be derived from the difference in threshold levels of H_2_O_2_ for the inactivation of various phosphatases, such as PTEN and PTP1B that target Akt and IR, respectively.

**Figure 10:**
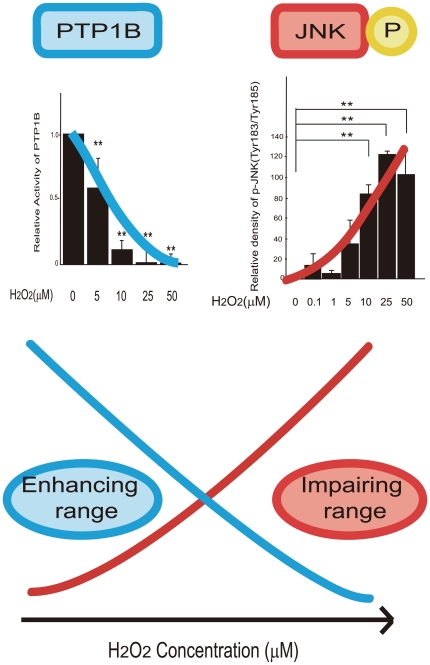
Dual action of H2O2 on insulin signal transduction can be explained by different thresholds for the repression of PTP1B activity and enhancement of JNK activation.

Our results identify JNK and phosphatases such as PTP1B as candidate molecules that determine the thresholds for the diametrical effects of H_2_O_2_ on cellular insulin signaling. We propose that the action of H_2_O_2_ on cellular insulin signaling is determined by the net balance between inactivation of PTP1B (the enhancive factor for insulin signaling) and activation of JNK (the inhibitory factor for insulin signaling) (Fig. 10). For example, 25–50 µM of H_2_O_2_ significantly decreased PTP1B activity, whereas these concentrations of H_2_O_2_ dramatically increased JNK phosphorylation simultaneously. As a result of the summation of these opposite two factors, insulin-induced Akt phosphorylation was impaired by 25–50 µM of H_2_O_2_ (Figure 2B), suggesting that the increase in JNK activation has a greater influence on net insulin signaling than the inactivation of PTP1B in these concentrations of H_2_O_2_. On the other hands, 10 µM of H_2_O_2_ induced a dramatic decrease in PTP1B activity and a moderate increase in JNK phosphorylation, resulting in an enhancement of insulin-stimulated Akt phosphorylation. These results suggest that the decrease in PTP1B activity has a greater influence than the increase in JNK phosphorylation in the cells treated with 10 µM of H_2_O_2_.

Early *in vitro* studies indicated that in the presence of H_2_O_2_, the pKa values and reaction rates for PTP1B were lower than those for TRX, an upstream regulator of JNK [25], suggesting that PTP1B may be more susceptible than TRX to oxidation by H_2_O_2_ *in vivo*. Thus, the dual actions of H_2_O_2_ on insulin signal transduction in H4IIEC hepatocytes may stem from the difference in susceptibility to H_2_O_2_ between PTP1B and TRX, although our study leaves open the possibility that other molecules in addition to PTP1B and TRX may participate in the dual effects of H_2_O_2_. Moreover, because we tested only the membrane-permeable ROS H_2_O_2_, we cannot exclude the possibility that factors other than concentration, such as intracellular localization, duration, or type of ROS, may influence the action of ROS on insulin signaling. Further investigations are required to elucidate the detailed molecular mechanisms underlying the dual effects of H_2_O_2_ on insulin signal transduction.

One limitation of our study is that our experiments were performed only in H4IIEC hepatocytes. At present, there are few reports demonstrating the dual action of ROS on insulin signaling in other cell lines. However, Loh et al. showed that mouse embryonic fibroblasts lacking Gpx1, an antioxidative enzyme involved in the removal of H_2_O_2_, exhibit enhanced insulin-induced Akt phosphorylation and PTEN oxidation, and normal tyrosine phosphorylation of the IR [8]. Thus, the positive regulation of insulin signaling by ROS may not be exclusive to H4IIEC hepatocytes. However, in the current study, we treated differentiated C2C12 myotubes with many concentrations of H_2_O_2_, but we could not find the dual effects of H_2_O_2_ on insulin signaling (data not shown). This result suggests that H_2_O_2_-induced dual effects on insulin signaling might be hepatocytes-specific, but additional studies on other insulin target cells such as adipocytes are needed to confirm that low concentrations of H_2_O_2_ enhance insulin signal transduction.

In conclusion, the current study demonstrates that depending on its concentration, H_2_O_2_ can exert either a positive or negative effect on insulin signal transduction in H4IIEC hepatocytes, and suggests that the intracellular ROS concentrations and different susceptibilities of signaling molecules to ROS are major determinants of impaired or enhanced insulin signaling. Further investigations into the dual effects of ROS on insulin signaling may lead to the development of novel antioxidative therapies to selectively combat the oxidative stresses that cause insulin resistance.

## Materials and Methods

### Materials

Antibodies against phospho-IGF-1 receptor (Tyr1131), phospho-insulin receptor (Tyr1146), insulin receptor β, Akt, phospho-Akt (Ser473), SAPK/JNK, phospho-SAPK/JNK (Thr183/Tyr185), phospho-GSK-3 (Ser21/9), and phosphor-serine636 of IRS-1 were purchased from Cell Signaling Technology (Beverly, MA, USA). Antibodies against GSK-3 and phospho-c-Jun were obtained from Santa Cruz Biotechnology (Santa Cruz, CA, USA). Human recombinant insulin was purchased from Sigma–Aldrich (St. Louis, MO, USA). Hydrogen peroxide, TiSO_4,_ xylenol orange, ammonium ferrous sulfate, and sorbitol were obtained from Wako Chemical Co., Ltd. (Osaka, Japan). PTP1B inhibitor came from Merck (Tokyo, Japan).

The rat hepatoma cell line H4IIEC was purchased from the American Type Culture Collection (Manassas, VA).

### Cell Harvesting and Western Blot Analysis

Cells were cultured in DMEM (Invitrogen, Carlsbad, CA, USA) supplemented with 10% FBS (Invitrogen), penicillin (100 U/mL), and streptomycin (0.1 mg/mL; Invitrogen) at 37°C in a humidified atmosphere containing 5% CO_2_. The cells were cultured to 60–70% confluence in 12-well plates. Next, the cells were serum-starved overnight and then treated with H_2_O_2_ for a total of 3 h, with medium changes every 30 min. After treatment, they were stimulated with recombinant human insulin (1 ng/mL) for 15 min, washed with ice-cold phosphate-buffered saline, and then lysed in RIPA buffer (Millipore, Billerica, MA) containing a Complete™ Protease Inhibitor Cocktail Tablet, EDTA-free (Roche Diagnostics) and PhosSTOP Phosphatase Inhibitor Cocktail Tablets (Roche Diagnostics). The lysates were sonicated using a Bioruptor sonicator (Cosmo Bio, Tokyo, Japan), and insoluble materials were removed by centrifugation. The resulting supernatants were separated by SDS-PAGE and transferred onto polyvinylidene difluoride (PVDF) membranes (Millipore). The membranes were blocked in a buffer containing 5% nonfat milk, 50 mM Tris (pH 7.6), 150 mM NaCl, and 0.1% Tween 20 for 1 h at room temperature and then incubated with specific primary antibody, followed by horseradish peroxidase-linked secondary antibody. Signals were detected using a chemiluminescence detection system (ECL Plus Western blotting detection reagent; GE Healthcare Bio-Sciences, Piscataway, NJ, USA). The membranes were subjected to direct densitometric analysis using a CCD camera system (LAS-3000 mini; Fuji-film, Tokyo, Japan) and Scion Image software.

### Measurement of H_2_O_2_ Concentrations in Culture Medium

Hydrogen peroxide concentrations in the culture medium were measured by ferrous oxidation of xylenol orange (FOX) assay [26]. Samples of culture media were added at specific intervals to FOX reagent, which comprised 100 µM xylenol orange, 250 µM ammonium ferrous sulfate, 100 mM sorbitol, and 25 mM H_2_SO_4_. Changes in absorbance at 560 nm were measured, and concentrations of H_2_O_2_ were calculated using a standard curve generated using H_2_O_2_ solutions of known concentrations.

### Preparation of H_2_O_2_

Hydrogen peroxide (30% v/v; Wako Chemical. Co., Ltd.) was diluted to a concentration of 100 µM in distilled water. The precise concentration of hydrogen peroxide was determined using the titanium oxide method [27], in which the molar coefficient of a titanium oxide-hydrogen peroxide complex is assumed to be 750 M^−1^ cm^−1^ at 405 nm. Briefly, 160 µl of hydrogen peroxide solution (prepared as described above) were added to a mixture of 30 µl of titanium sulfate and 50 µl of 20% (v/v) hydrogen sulfate. The resulting mixture was stirred at room temperature for 15 min, and the precise concentration of hydrogen peroxide was calculated from the absorbance at 405 nm.

**siRNA transfection in H4IIEC hepatocytes.** H4IIEC hepatocytes were transiently transfected with 30 nM of siRNA duplex oligonucleotides using Lipofectamine RNAiMAX (Invitrogen) according to the manufacturer's instructions, as described previously [28]. A JNK1-specific siRNA was synthesized by Dharmacon (USA): 5′-GAAUAGUGUGUGCAGCUUA-3′ (sense). Negative control siRNA was purchased from Dharmacon. One day after transfection, cells were stimulated with 1 ng/ml of human recombinant insulin for 15 min.

### Statistical Analysis

Differences between two groups were tested by unpaired, two-tailed Student's *t*-tests. More than two groups were compared by one-way analysis of variance (ANOVA). All calculations were performed using StatView software (SAS Institute Inc., Cary, NC, USA).
